# Magnetophoretic Decoupler for Disaggregation and Interparticle Distance Control

**DOI:** 10.1002/advs.202100532

**Published:** 2021-05-01

**Authors:** Hyeonseol Kim, Byeonghwa Lim, Jonghwan Yoon, Keonmok Kim, Sri Ramulu Torati, CheolGi Kim

**Affiliations:** ^1^ Department of Emerging Materials Science DGIST Daegu 42988 Republic of Korea

**Keywords:** bead pair, decoupler, disaggregation, magnetic field, magnetophoresis, wave‐like pattern

## Abstract

The manipulation of superparamagnetic beads has attracted various lab on a chip and magnetic tweezer platforms for separating, sorting, and labeling cells and bioentities, but the irreversible aggregation of beads owing to magnetic interactions has limited its actual functionality. Here, an efficient solution is developed for the disaggregation of magnetic beads and interparticle distance control with a magnetophoretic decoupler using an external rotating magnetic field. A unique magnetic potential energy distribution in the form of an asymmetric magnetic thin film around the gap is created and tuned in a controlled manner, regulated by the size ratio of the bead with a magnetic pattern. Hence, the aggregated beads are detached into single beads and transported in one direction in an array pattern. Furthermore, the simultaneous and accurate spacing control of multiple magnetic bead pairs is performed by adjusting the angle of the rotating magnetic field, which continuously changes the energy well associated with a specific shape of the magnetic patterns. This technique offers an advanced solution for the disaggregation and controlled manipulation of beads, can allow new possibilities for the enhanced functioning of lab on a chip and magnetic tweezers platforms for biological assays, intercellular interactions, and magnetic biochip systems.

## Graphical abstract

This study reports the real‐time disaggregation and interdistance control of the magnetic beads using magnetophoretic decoupler fabricated with permalloy thin film pattern. The application of a rotating magnetic field creates a unique magnetic potential energy distribution around the decoupler at multiple points, and thus, separates the aggregated beads and transported them on the pattern to the desired direction.

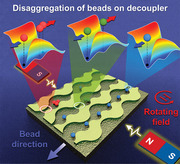

## 1 Introduction

The controlled manipulation of magnetic beads or cells is recognized to be of significant importance for both fundamental and applied research, such as biomedical, diagnostics and therapeutics, drug delivery, and single‐cell analysis.^[^ ^1^, ^2^, ^3^, ^4^, ^5^ ^]^ In a magnetophoretic platform, the target cell or protein labeled with a magnetic bead is controlled based on the magnetic force generated by the interaction between the magnetic bead and the external magnetic field using a magnet or coil outside the platform environment, where the carrier beads can be controlled easily without physiologically damaging the target cells or proteins.^[^ ^6^, ^7^, ^8^ ^]^ For example, it is possible to effectively separate nonmagnetic cells and move them to the desired position individually. This allows analysis at the single‐cell level, providing the heterogeneities in single‐cell properties with its population level.^[^ ^9^ ^]^

Alternatively, the energy landscape induced particle motion caused by applying the rotating magnetic field was used for colloidal particles, large molecules, and cell transportation and separation along the surfaces of the substrate, patterned magnetic surfaces, integrated circuits of overlaid patterns of magnetic films, and garnet films.^[^ ^7^, ^10^, ^11^, ^12^ ^]^ However, the irreversible aggregation or clustering of original carrier beads owing to their magnetic interactions is indeed a critical and limiting factor in the individual transportation and separation processes.^[^ ^13^ ^]^ This aggregation issue may prevent the quantification of the physical and biological properties of various DNA, proteins, or cells because of the gap between objective beads.^[^ ^14^, ^15^, ^16^ ^]^ In particular, the particles were transported and sorted when they were not aggregated before performing the manipulation. However, these reports do not describe thoroughly the process of manipulation or real‐time disaggregation and separation of particles that are already aggregated in a pattern. Although the aggregation can be eliminated temporarily using the z‐field, reaggregation occurs eventually when the in‐plane magnetic field is again applied to control the beads during separation.^[^ ^17^, ^18^ ^]^ In fact, the aggregation of multiple beads occurs because the energy well created by the external field is larger than the nano‐microbeads used for labels and thus multiple beads gather at a single energy well.^[^ ^18^, ^19^ ^]^ Here, since the size of the potential energy well created by bulk magnets or magnetic coils is too large to confine a single bead, the interparticle distance of trapped beads cannot be controlled individually.^[^ ^20^, ^21^ ^]^

In general, the constrained domain walls in ferromagnetic nanoconduits patterned on a substrate can be used for manipulating magnetic nanocarriers on‐chip by tuning the size of the domain walls from a few tens of nanometers wide up to micrometers, where the resulting trapping potential well is of similar size, which prevents or mitigates aggregation and allows manipulation of individual nanocarriers.^[^ ^22^, ^23^ ^]^ However, the potential well is created above the domain wall where different magnetizations meet for the control of the particle, resulting in several issues. For example, if the size of the conduit is increased to control the larger size particle, the domain wall does not scale up accordingly, and thus, becomes form a complex multidomain. As a result, the potential well is also not uniform. And, using the movement of the single domain wall along with the pattern only one particle per conduit can be manipulated.^[^ ^22^ ^]^ Although, using *z*‐field single particle can be controlled more precisely by an array of conduits but the *z*‐field applied for the transfer of the particle between each component also affects the potential well of all the particles located on the chip and thus it is difficult to control multiple particles parallelly.^[^ ^22^, ^23^ ^]^

Recently, combined technologies have been used for controlling the space between driving particle and fixed particle for biological applications. However, if the driving particle is magnetic, then the fixed particle has to be controlled by other methods, i.e., micropipette, acoustic and optical tweezing, and or hydrodynamic traps, which required additional processing and equipment.^[^ ^15^, ^24^, ^25^, ^26^ ^]^ Also, magnetic tweezers require an additional parallel tip for precisely controlling each bead; otherwise, the beads present in the total focusing area are attracted in the same direction and aggregate at one point.^[^ ^27^, ^28^ ^]^ Therefore, it is necessary to develop a reliable 2D magnetic platform for controlling the beads on a large scale for practical magnetic tweezers platforms without any aggregation. Moreover, achieving accurate manipulation and disaggregation of particles on a 2D platform with controllable interparticle distance is crucial for monitoring the interaction dynamics between cells in on‐chip devices. To the best of our knowledge, there is no available micromagnet pattern platform that achieves both the disaggregation of beads with individual control and for interparticle distance control.

Here, we propose a magnetophoretic decoupler pattern that achieves efficient magnetic bead disaggregation and interparticle distance control by applying an external magnetic field with a uniform rotational speed. The magnetic pattern was made of permalloy using conventional photolithography and lift‐off processes. The aggregated magnetic beads were consecutively detached into single ones by the successive splitting and delay of the magnetic potential wells owing to the specific shape of the magnetic patterns, demonstrating the simultaneous and accurate spacing control of multiple magnetic bead pairs. Hence, the proposed magnetic method could be a milestone achievement on the magnetic tweezers platform for performing biological interactions and extracting relevant information at the level of a single object.

## 2 Results and Discussions

### 2.1 Conceptual Design, Manipulation Principle and Optimization

In the magnetophoretic transportation system, the magnetic beads or magnetic fluid droplets were manipulated using the angular variation of the magnetic potential energy distribution, which is generated according to the 2D shape of the magnetic thin film under a rotating magnetic field. If the applied rotating magnetic field is in the in‐plane direction, the magnetic bead around the thin film disk slides along the boundary of the disk. This brings into focus the motion of a magnetic bead between thin films of different size of disks. The two factors that can affect the movement of the bead are the bead and pattern size, and the gap between the patterns. Based on the relative size of the bead and patterns, there could be two possible modes for the movement of the bead between the two disks in following one of the disk boundaries.

**Figure 1:**
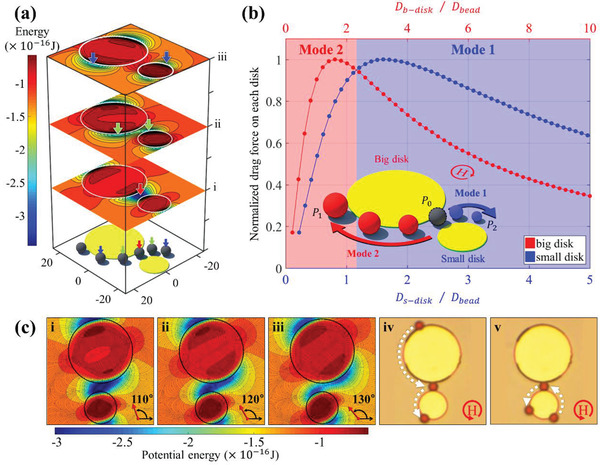
Proof of concept for “mode” switching of the bead: a) Magnetic potential energy distribution around the two disks under the rotating magnetic field of 90°, 130°, and 170°. The magnetic beads move along the disk boundary along with the direction of the magnetic field owing to the location of the potential energy minima, as shown in blue. Here, the potential minima move during field rotation, where the beads located (colored arrows) split into two and moves in the opposite direction along with each disk (i–iii). b) Change of the drag force according to the ratio between the diameter of each disk (red: big disk and blue: small disk) pattern and the bead. The big disk is fixed at twice size of the small disk ( *D*_b − disk_ =  2  × *D*_s − disk_). When the small disk is more than 1.2 times the bead diameter, the beads moves toward the smaller disk, otherwise they move towards the big disk. The inset illustrates the two paths for bead movement when the beads were placed at the initial position (*P*_0_) between the different sizes of disk (DSD) patterns (“Mode 1” to *P*_1_ and “Mode 2” to *P*_2_). c) Angular variation of the magnetic potential energy for the disk patterns with diameters of 15 and 30 µm with a 4 µm gap for 4.5 µm bead movement. ci–iii) Detailed angular variation of the simulated potential energies. The overlapped minimum energy for the two disks is split into two minima from a field angle of 130°, where the energy barrier is formed near big disk. Thus, the bead moves to the small disk (the dark blue represents the lower energy). civ,v) Because the small disk's size ratio (*D*_s − disk _/*D*_bead _ =  3.33), according to the graph in (a), the bead always experiences a large attractive force towards the small disk irrespective of their initial starting point. civ) Experimental observation showing motion in the “Mode 1" during which the beads starting from the big disk are directed to the small disk. cv) Experimental observation showing a motion in “Mode 1” during which a bead starting from the small disk is directed back to it.

As seen in **Figure** **1**a, when the magnetic field rotates (i to iii), the potential energy well splits and then moves away in both directions along the boundaries of the small and big disks, where the bead can be located (white circle). Hence, to understand the two modes for the direction of motion of the beads, we compared and normalized the magnitude of the simulated magnetic forces on the beads caused by the disks of different diameters (Figure 1b). At this time, the disk is assumed to be twice the size of the small disk ( *D*_b − disk_ =  2  × *D*_s − disk_). It can be noted that the magnetic force increases with the bead size and reaches a maximum when the diameter of the small disk is ≈1.2 times the diameter of the bead and then decreases as the disk diameter increases further. When *D*_s − disk _/*D*_bead _ = 1.2 (*D*_b − disk _/*D*_bead _ = 2.4), the forces of the small and disks attain the same value. Based on these simulations, the bead is expected to move along the boundary of the small disk, “Mode 1,” if the diameter of the small disk exceeds 1.2 times the bead diameter (*D*_s − disk _/*D*_bead _ ≥ 1.2, Movie S1, Supporting Information). However, it moves along the boundary of the big disk, “Mode 2,” when the diameter of the small disk is less than 1.2 times that of beads (*D*_s − disk _/*D*_bead _ ≤  1.2, Figure 1b and Movie S1, Supporting Information). Considering the accuracy of the fabrication pattern, we focus on the “Mode 1” switching phenomenon in this study.

For a bead whose diameter is 4.5 µm, the angular variation of magnetic energy and the direction of magnetic drag force on the permalloy disks of diameters 15 µm (*D*_s − disk _/*D*_bead _ = 3.33) and 30 µm (*D*_b − disk _/*D*_bead _ = 6.66) with varying angles from 110° and 130° at 120 Oe of external magnetic field is shown in Figure 1ci–iii. The dark blue color represents a lower energy distribution. From 110° to 120°, the energy minimum of each disk overlaps and continues to be maintained as a single well. Subsequently, it splits into two minima as it reaches 130°. At this time, a potential barrier is formed between the two minima. The minimum energy point of the small disk is closer to the position before separation compared to the big disks. Thus, the bead remains near the smaller disk and then moves continuously along the minimum energy point toward the boundary of the small disk. In particular, we observed that the bead ultimately follows the boundary of the small disk after the gap between the two disks, regardless of the initial position of the bead, as confirmed by the experimental results in Figure 1civ,v (Movie S1, Supporting Information).

When multiple beads are located on the gap, the local minimum energy of beads is different owing to their spatial distribution, and thus the aggregated beads can be detached by the movement of the split local energy minimum. Based on the separation of two energy minima during the field rotation, it is possible to detach the aggregated beads, send them to one disk, and make them rotate at the boundary of the occupied disk. To move the bead in only one direction after disaggregation, we extended the different size disk (DSD) pattern to a wave‐like DSD pattern because there is no significant difference in the potential energy variation between the disk and wave patterns for the calculated energy, where the wave‐like DSD pattern allows the bead to move along with the pattern (Figure S1 and S2, Supporting Information).

**Figure 2:**
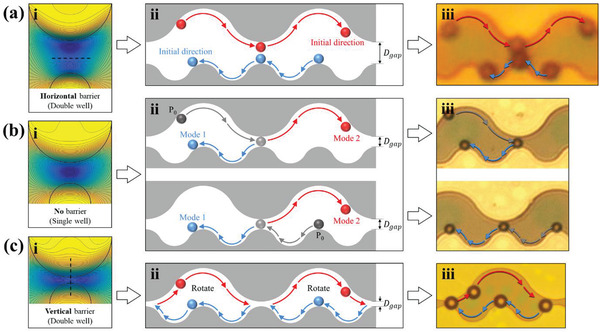
Different movements of the bead according to the form of the controlled potential barrier by adjusting the gap size: If a horizontal (*D*_gap_ */D*_bead _ > 1.4) or vertical (*D*_gap_ */D*_bead _ < 0.8) barrier is formed between wells, the bead does not exhibit “mode” switching. ai) In the case of the horizontal barrier, the beads at each wave pattern moves without “mode” switching. aii) Schematic of the movement of the bead on a wave‐like pattern. aiii) Good agreement between the experimental observations and simulated results. The red and blue arrows represent the directions of the bead movement. bi) “Mode” switching occurs only if there is a single well without a potential barrier (*D*_gap _/*D*_bead _ =  0.8  − 1.4). bii) Schematic of the “mode” switching of beads on a wave‐like pattern, where “*P*_0_” is the initial position of the bead. biii) Experimental observations of “mode” switching is consistent with simulation results. The red and blue arrows represent the directions of the movement of the bead. ci) In the case of a vertical barrier, the bead cannot cross the gap, is trapped, and rotates repeatedly in the track in which it is trapped. cii) Schematic representation of bead trapping and rotation on the wave‐like pattern. ciii) Experimental confirmation of bead trapping and rotation on the track.

Depending on the gap size (second factor), potential barriers are created or annihilated in different directions. If the gap is larger than 1.4 times the bead diameter (*D*_gap _/*D*_bead _ > 1.4), a horizontal potential barrier is created between two potential wells of patterns. Thus, the beads in each wave boundary cannot switch from one to another (**Figure** **2**ai–iii and Movie S2, Supporting Information) and no “mode” switching occurs by maintaining the initial direction of travel. “Mode” switching is possible only if there is a single well is formed without a potential barrier, i.e., *D*_gap _/*D*_bead _ =  0.8 −  1.4 (Figure 2bi–iii and Movie S2, Supporting Information). However, if the gap is less than 0.8 times the bead diameter (*D*_gap _/*D*_bead _ < 0.8), there is still no “mode” switching because of the vertical barrier, and thus the bead cannot cross the gap and move to the next pattern (Figure 2ci–iii and Movie S2, Supporting Information). Hence, the bead shows the switching phenomenon by moving along the boundary of a particular wave, depending on its size only in the above range (*D*_gap _/*D*_bead_ =  0.8 − 1.4). Thus, the bead moves only in one direction under one size ratio condition.

For continuous operation on 2D connected structures, beads should be sent from the gap to the big wave by the Mode 2 or Mode 1 (in the case of detached bead from the disaggregation process) for further process on the next gap. However, the strength and frequency of the field play a key role for the movement of the bead on the big wave (Figures S3 and S4a, Supporting Information). In order to find an optimized condition for the smooth movement of the bead on a big wave and transported to the next gap, the strength and frequency of the field were changed in such way that the difference in force generated by each wave was sufficiently significant (*D*_s − wave _/*D*_bead _ =  3.57, Figure 1b). When the field strength exceeds 20 Oe, the bead on the big wave was able to move to the next gap at lower frequencies (Figure S4b and Movie S3, Supporting Information). However, in the higher frequency range, the more discontinuous movement is caused by the resistance of the fluid. Therefore, to reduce the uncertainty effects of the fluid resistance, we use the lowest frequency with certain field strength that only repeat the reproducibility of the bead movement in each time (Figure S4, Supporting Information). As a result, the optimized field strength of 120 Oe with a frequency of 0.05 Hz was used for smooth movement of the bead.

**Figure 3:**
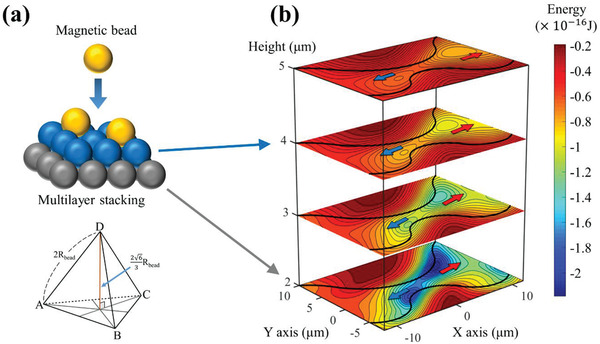
Potential energy distribution according to the vertical distance assuming that beads are stacked in multilayer: a) Concept of beads being stacked in a multilayer. In the absence of a magnetic field, the beads are stacked in the form of a tetrahedron, and the distance between the first and second layers is $\frac{2\sqrt{6}}{3}R_{\mathrm{bead}}$. The vertical height of the bead on the first layer is the sum of the radius of the bead (*R*_bead_) and the thickness of the passivation layer (*t*_p_). The vertical height of the next layer should be the sum of the first layer and the distance between layers ($\frac{2\sqrt{6}}{3}R_{\mathrm{bead}}$). b) Potential energies for bead layers with different vertical height. The depth of the energy well varies depending on the center of the bead at each layer, but it can be seen that wells are created and moved in opposite directions around the gap irrespective of vertical height. Hence, disaggregation can be achieved regardless of the vertical height because eventually, only a single layer structure or single bead remains on the 2D surface.

Furthermore, if the beads were stacked in layers to form a 3D structure rather than a 2D one or if the thickness of the protective layer under the bead was different and the center of the bead was located at a certain vertical distance, the bead would be located at different potential energy distributions depending on the distance (**Figure** **3**). However, since the in‐plane magnetic fields used in this study were sufficiently strong and continuously changing, as a result, most of the effective fields on each bead are parallel to the surface, and the magnetic moment of each bead is in the same direction. Hence, the magnetic beads, which are dispersed in 3D in a liquid, fall to the surface owing to gravitational and magnetic forces. Consequently, they do not stack up with each other owing to the magnetic repulsive force via dipole–dipole interaction and also because of the location of the ever‐changing bottom bead. However, it can be made into a multilayer by deliberately stacking through sinking without applying a magnetic field (Figure 3a). The core operating principle, i.e., the potential well moving in different directions at the gap has different potential well depths, works in the same way for few layers with vertical distance (Figure 3b). Eventually, only a single‐layer structure or a single bead remains in the gap between the patterns. Hence, all the experimental results were satisfied the condition that the beads were in a single layer and used the optimized experimental parameters of field strength of 120 Oe and frequency at 0.05 Hz for accurate bead movement. Here, depending on the direction of the detached beads, two types of applications of decoupling (condition is highlighted in green in Tables S1 and S2, Supporting Information) and interparticle distance control (condition is highlighted in blue in Tables S1 and S2, Supporting Information) are possible, as discussed below.

### 2.2 Decoupling and Switching of Magnetic Beads in an Array

**Figure 4:**
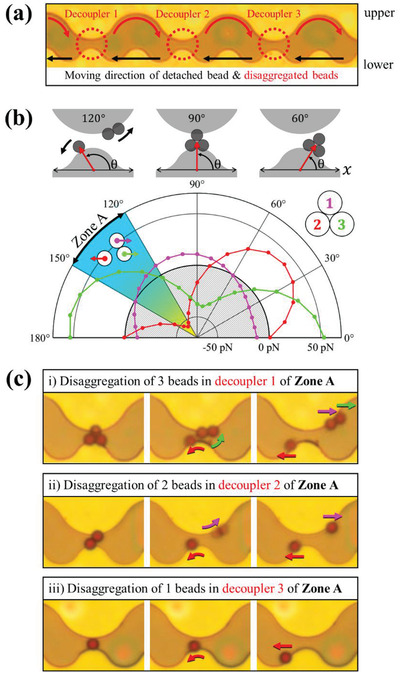
Disaggregation and movement of bead near decoupler with wave‐like pattern: a) Movement of disaggregated single beads and aggregated beads on the wave‐like patterns for different sizes and gaps of the decoupler, where the beads can go to the next decoupler. b) Angular dependence of the simulated forces of aggregated beads. The beads are clustered at one point due to a potential well between field angles of 60° and 120°. It is assumed that the magnetic moment of each bead rotates along the external magnetic field, and the *x*‐component of the magnetic force due to the potential energy generated at each bead position indicates that the bead *2* (with negative force) moves to the left (−*x*‐direction) and bead 1 and bead 3 (with positive force) moves to the right (+ *x*‐direction) in angle zone A. c) The sequential disaggregation process of aggregated beads into single beads. i) Disaggregation of single from clustered beads and motion in the −*x*‐direction for single beads along the lower track and in the + *x*‐direction along the upper track for two beads. ii) Disaggregation of individual beads and motion in the −*x*‐direction along the lower track (“Mode 1”) and in the + *x*‐direction along the upper track (“Mode 2”). iii) A single bead crosses the gap (decoupler 3) and eventually navigates in the −*x*‐direction.

According to the analysis of switching modes, it is more convenient to adjust the interbead forces for the performance as a decoupler, that is, decoupling and switching in one direction. **Figure** **4** shows the calculated interbead forces during the rotation of the field and an experimental demonstration of decoupling and the subsequent movement in the wave‐like DSD pattern. In Figure 4a, the two half‐disks facing each other function as a decoupler with different gaps between them. The lower pattern also functions as a moving track for the bead in the − *x*‐direction after they decoupled under the rotating field in a counter‐clockwise direction. Figure 4b shows the schematics of the aggregated three beads’ positions for field angles of 60°, 90°, and 120° (upper figure), and the polar plot of the *x*‐component of the calculated forces as a function of the field angle (lower figure). The magnitude of the force (*F*) is approximately tens of pN, which is dominant compared to the dipole–dipole interaction (≈several pN) between each bead. Here, the ± sign of the force indicates that the force acts in the ± *x*‐direction, as a function of the field angle. When the origin of the position (*P*_1_, *P*_2_, and *P*_3_) of the bead is arranged as a triangle from the center of the gap, the calculated forces *F*_1_, *F*_2_, and *F*_3_ of each bead labeled 1, 2, and 3 are as follows

(1) $$\begin{array}{ccc} & & \mathrm{At}\;\mathrm{the}\;120^{\circ }\leq \theta \leq 150^{\circ },\\ & & F_{n}\left(\theta \right)\left\{\begin{array}{cc} \mathrm{Bead}\;\;1:F_{1}\left(\theta \right)>0, & P_{1}\left(0,+R_{\mathrm{bead}}\times \;2\tan30^{\circ }\right)\\ \mathrm{Bead}\;\;2:F_{2}\left(\theta \right)<0, & P_{2}\left(-R_{\mathrm{bead}},-R_{\mathrm{bead}}\times \tan30^{\circ }\right)\\ \mathrm{Bead}\;\;3:F_{3}\left(\theta \right)>0, & P_{3}\left(+R_{\mathrm{bead}},-R_{\mathrm{bead}}\times \tan30^{\circ }\right) \end{array}\right. \end{array}$$

Even though the acting force directions for the beads are different for *θ* < 90°, the three beads move together because no bead reached to the gap. When *θ* = 90°, the beads reach the decoupler. When angle 120°≤ *θ* ≤150° (Angle zone A), the forces of beads 1 and 3 are positive, but that of bead 2 is negative. This means that beads 1 and 3 move in the +*x*‐axis direction along the upper track while bead 2 moves in the − *x*‐direction. For a further rotating field, bead 2 navigates in the − *x*‐direction, but beads 1 and 3 reach the decoupler 2 moving along the + *x*‐direction after the half‐cycle of the rotating field. At the decoupler 2, two beads become new beads 1 and 2 and splits in opposite directions at the same field angle of zone A. In fact, the N‐ and S‐magnetic poles are topologically the same after rotation of the half‐cycle of the field. Therefore, we calculated only the first half‐cycle. The subsequent rotation repeats the same movement of the potential energy as the first half. During further rotation of the field, a single bead from decoupler 2 moves in the + *x*‐direction (upper track) and arrives at decoupler 3. Here, bead 3 switches to a lower track of a smaller wavy pattern and starts to move in the − *x*‐direction along the lower track (Figure 4ci–iii).

**Figure 5:**
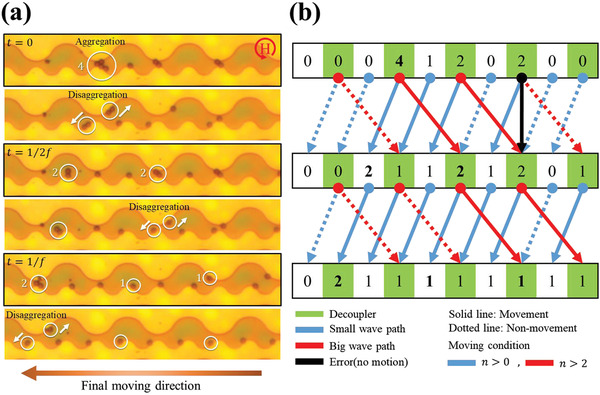
Directional movement of single magnetic beads on the wave‐like pattern: a) The aggregated magnetic beads are split into single beads by the serial decoupler and then eventually move in the −*x*‐direction. The detached beads move to the adjacent decoupler in time intervals of $\frac{1}{2f}$, $\frac{1}{f}$, etc. b) Change in the number of beads at the crest of a small wave at *t*  =  0, $\frac{1}{2f}$, $\frac{1}{f}$, that is, after integers of half cycles of the field. The green color indicates the gap of the decoupler where the two waves meet. The beads are split in the decoupler and all the single beads move only in the −*x*‐direction under the rotating magnetic field. The number in bold on the diagram is the disaggregation process of the beads tracked by a white circle in Figure 5a. (The condition is highlighted in green in Table S2, Supporting Information.)

**Figure** **5** shows the serial decoupling of multiple beads and the switching to the lower track in the serial decoupler arrays (Movie S4, Supporting Information). Here, the decoupling of *n*‐multiple beads requires (*n* − 1) decouplers with gaps because decouplers divide the beads in the cluster into two groups. Each decoupling further divides the cluster and finally detached a single bead after (*n* − 1) decoupling. Further, one more decoupler is required to switch the “mode” of the last detached single bead, and thus, a total of *n* decouplers are required for single bead disaggregation and transfer (Figure 5a). Figure 5b shows a series of processes as a numerical diagram for disaggregating the beads during the (*n* − 1) integer of half‐cycle of field. The decoupler, where two waves meet, is marked in green color. The bold number represents the disaggregated beads on the decoupler, as shown in Figure 5a. The aggregated beads, which are detached only in the decoupler, continue to move towards the next decoupler along the upper track until a single bead remains in the decoupler. When a single bead comes to the decoupler, it switches to the lower track and moves in the − *x*‐direction. Hence, this concept can clarify the bead aggregation that inevitably occurs in various experiments using magnetic bead carriers and can be controlled locally without additional tools.

### 2.3 Interparticle Distance Control of a Pair of Magnetic Beads

**Figure 6:**
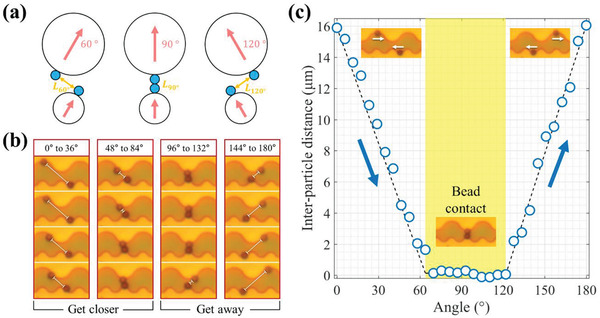
Interparticle distance control during the aggregation and disaggregation of pairs of magnetic beads: a) Schematic representation of the adjustment of the bead spacing by changing the field angle of applied field from 60° to 120° (indicated in blue in Table S2, Supporting Information). b) Bead translocation along the wave‐like pattern when the external magnetic field rotates from 0° to 180°. c) Change in the interparticle distance between beads was measured for the angle change of 5.81°. The distance is linearly adjusted except for the areas where the beads are in contact with each other (angle from 61.88° to 118.13°). The experimental results and calculations are in good agreement, confirming that the potential well begins to go away from 120°.

Under the nonswitching “mode” condition, interbeads distance can be adjusted depending on the field angle (**Figure** **6**a). Figure 6b shows that the two beads of each wave boundary move in opposite directions depending on the angle of the external magnetic field. It can be seen that the interbead distance between them is adjusted linearly. The two beads are in contact with each other when the field angle lies between ≈60° and 120°, and also rotates in the opposite direction (clockwise) of the field along the potential well where each bead is located . This indicates that the bead movement is dominated by the potential energy gradient created by the pattern rather than the dipole–dipole interaction. The spacing between the beads in the angle range 0°–60°, where the beads are approaching, changes almost linearly from 16 to 0 µm (Figure 6c). However, when the field angle exceeds 120°, the beads move away from each other. In other words, if a magnetic field is applied at a specific angle using a bulk magnet, the two beads can be maintained at a certain distance semi‐permanently. If these patterns are repeated on a 2D surface, it is also possible to maintain several bead pairs at the same interparticle distances for a long time (Figure S5a and Movies S5 and S6, Supporting Information).

### 2.4 Extensions to Nonmagnetic Bead Disaggregation

**Figure 7:**
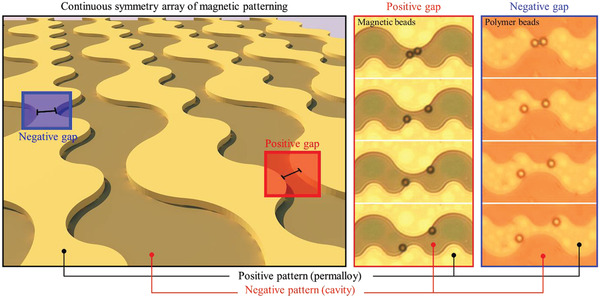
Selective control of magnetic/nonmagnetic beads by dual positive and negative magnetophoretic patterns: After filling the outer space of the 2D surface pattern with magnetic fluid, the condition for negative magnetophoresis is satisfied to reproduce all the preceding experiments for nonmagnetic polymer beads. For the simultaneous and selective control of magnetic beads and polymer beads, the surface patterning was designed so that the magnetic pattern and the cavity shape were repeated continuously on the 2D surface and vertically symmetric with each other. This design has affirmed the simultaneous disaggregation of aggregated magnetic and nonmagnetic beads under a rotating magnetic field in the same direction and presents the possibility of moving a single bead in different directions. However, in this case, the polymer beads could be moved only under the high external field of 900 Oe at 0.2% of ferrofluid concentration, unlike the magnetic beads designed to operate at 120 Oe.

The proof of concept for magnetic beads with positive magnetophoresis could be extended to nonmagnetic particles using a negative magnetophoresis technology with magnetic fluid^[^ ^29^, ^30^ ^]^ (**Figure** **7** and Figure S5b, S6 and S7 the conditions are highlighted in green in Tables S3 and S4, Supporting Information). As seen from Figure S6 in the Supporting Information, the aggregated polymer beads whose diameters were 6.72 and 11 µm on a wave pattern were split into single beads by a decoupler of negative pattern with an appropriate gap (6 µm) by applying an external field of 900 Oe and they moved in one direction after switching from the upper to the lower track. Even if the pattern size was fixed, the polymer bead with a diameter of 11 µm behaved differently because of the size ratio (Movie S7, Supporting Information). For the two types of beads with diameters of 11 and 6.72 µm, the ratios of the gap to the bead (*D*_gap _/*D*_bead _) are 0.54 and 0.89, respectively. However, according to Figure 2c, the “mode” switching range is revealed in the range of 0.8− 1.4. Hence, only the 6.72 µm beads pass the gap, whereas the 11 µm beads only rotate, as the *D*_gap _/*D*_bead _ for the 11 µm beads lies beyond the “mode” switching range to be trapped.

Micromagnetic patterns can be designated to possess dual (positive and negative) magnetophoretic functions because the positive magnetic pattern causes a negative cavity pattern, as shown in Figure 7. In other words, the 2D surface patterning includes a positive gap made of permalloy for positive magnetophoresis (red box) and a negative gap made of a cavity for negative magnetophoresis (blue box). The magnetic pattern (wave pattern) and cavity patterns are arranged in vertical symmetry to each other. After filling the outer space with a low‐concentration magnetic fluid that does not affect the properties of biological entities such as proteins and cells, it is possible to control the nonmagnetic polymer beads, similar to the manipulation of magnetic beads by utilizing the cavity as a negative magnetic pattern.^[^ ^10^, ^31^ ^]^ Thus, it is possible to move the polymer beads in the opposite direction (right side as in Figure S7, Supporting Information) under the same rotating magnetic field (counter clockwise) . Similar to the case of magnetic beads, the interparticle distance can be adjusted for the polymer beads, as shown in Figure S5b in the Supporting Information. Overall, the developed technique succeeds to perform the simultaneous disaggregation and interparticle distance control (Movie S6, Supporting Information).

## 3 Conclusions

We have demonstrated a magnetophoretic technology for the decoupling and switching of magnetic beads using 100 nm thick magnetic patterning and an external magnetic field. This decoupler is realized by a unique magnetic potential energy distribution in the form of an asymmetric magnetic pattern with a nonmagnetic gap, where the decoupling performance of magnetic/nonmagnetic beads is demonstrated according to carrier size and pattern. The proposed disaggregation technique offers new possibilities for various applications, including magnetic tweezers and lab‐on‐chip processes, where the advantage of a uniform particle size configuration can be integrated with the biological assay to control the positioning of cells for studying the intercellular interactions solving the issue related to the irreversible aggregation of beads used as labels for various studies.

As most of the existing methods can control only a pair of beads at a time in turns,^[^ ^32^, ^33^, ^34^ ^]^ it takes a lot of effort to repeat the process for stable results. In contrast, the proposed method can simultaneously manipulate multiple bead pairs in the 2D space with the same force and spacing, thereby reducing the time‐consuming steps to perform several sets of experiments. Here, controlling the distance of a bead pair from multiple points is accomplished easily through the angles of an external magnetic field. In addition, depending on the direction or the size of the decoupler installed in one plane, the beads located on each decoupler are adjusted in different directions and distances. However, a few limitations exist in the proposed technique, such as, if the surface is required an additional layer of thickness for immobilizing biomolecules, there is a reduction of the force on the magnetic beads depending on the thickness. Then, the difference in operating the magnetic field strength for both beads increases for the dual positive and negative magnetophoretic platforms.

## 4 Experimental Section

##### Materials for Positive and Negative Magnetophoresis

Superparamagnetic beads (M‐280 Streptavidin, M‐450 Tosylactivated from Invitrogen, Grand Island, NY, USA) and nonmagnetic polymer beads (Aminopolystyrene beads, AP‐35‐10, AP‐60‐10, SPHERO) were used in this experiment. In the experiments, the magnetic beads were diluted to 0.001% with distilled (DI) water and nonmagnetic beads were added to water‐based ferrofluid (EMG 700, Ferrotec Co., Bedford, NH) in a ratio of 0.01% to the total volume. Ferrofluid was diluted with DI water at a concentration of 0.2%. Photoresist (AZ‐5214E) and developer (AZ‐300 MIF) were purchased from Merck Performance Materials, GmbH. The Si wafer was coated with SiO_2_ layer of 500 nm thickness. Parylene C (DiX‐C from KISCO) was used to coat the surface of the pattern.

##### Magnetophoretic Surface Patterning and External Magnetic Fields

The fabrication process for detailed magnetophoretic surface patterning was described in the previous articles.^[^ ^35^, ^36^ ^]^ In brief, Ni_80_Fe_20_ (100 nm thickness) was deposited on the substrate in a specific shape through photolithography and DC magnetron sputtering. Then, to protect the pattern in the fluid environment and reduce the adhesion force between the bead and the substrate, parylene C (500 nm thickness) was coated with chemical vapor deposition. The external rotating magnetic field was implemented in two ways depending on its strength. Two perpendicular solenoid coils produced a rotating magnetic field of 0–200 Oe within the *x*–*y* plane.^[^ ^37^ ^]^ For nonmagnetic polymer bead manipulation, the required strong field strength of 900 Oe was supplied by a rotatable bulk magnet with adjustable spacing between each other. The rotational speed of this magnet was determined by the motor and the strength of the magnetic field can be controlled by adjusting the spacing.

##### Magnetic Simulation

Micromagnetic domain simulations were performed using MuMax^3^ software. Standard material parameters such as exchange stiffness *A*_ex_ = 1.0 × 10^−11^ J m^−1^, saturation magnetization *M*_s_ = 800 kA m^−1^, damping constant *α* = 0.1, zero magnetocrystalline anisotropy, and cell size of 100 × 100 × 100 nm^3^ were used for simulation. Using the stray field created by the simulated magnetic domain, the distribution of the magnetic potential energy and magnetic force exerted on the bead was calculated using a Matlab code. The distance from the surface at which the magnetic forces are calculated for a single bead or a single layer of 2D aggregated beads is equal to the sum of the radius of the bead and the thickness of the passivation layer (Parylene, 0.5 µm). All the conditions were calculated and analyzed based on the above vertical height.

## Conflict of Interest

The authors declare no conflict of interest.

## Supporting information

Supporting Information

Supplemental Movie 1

Supplemental Movie 2

Supplemental Movie 3

Supplemental Movie 4

Supplemental Movie 5

Supplemental Movie 6

Supplemental Movie 7

## Data Availability

Research data are not shared.
