# Supplementary material for: Magnetophoretic Decoupler for Disaggregation and Interparticle Distance Control
Source: Adv Sci (Weinh). 2021 May 1;8(12):2100532. doi: 10.1002/advs.202100532 (PMC8224445; doi:10.1002/advs.202100532)
Supplement: Supplementary file 1 — Supporting Information [file ADVS-8-2100532-s007.pdf]

© 2021 Wiley-VCH GmbH

ADVANCED SCIENCE
Open Access

Supporting Information

for *Adv. Sci.,* DOI: 10.1002/advs.202100532

Magnetophoretic decoupler for disaggregation and inter-particle distance control

Hyeonseol Kim, Byeonghwa Lim, Jonghwan Yoon, Keonmok Kim,

Sri Ramulu Torati*, CheolGi Kim*

Supporting Information

Magnetophoretic decoupler for disaggregation and inter-particle distance control

Hyeonseol Kim, Byeonghwa Lim, Jonghwan Yoon, Keonmok Kim, Sri Ramulu Torati*, CheolGi Kim*

Department of Emerging Materials Science, DGIST, Daegu, 42988, Republic of Korea.

*Corresponding authors: E-mail: srtorati@dgist.ac.kr (SR Torati), cgkim@dgist.ac.kr (CheolGi Kim), Tel: +82-53-785-6516, Fax: +82-53-785-6509

Tables

Table S1. Condition for decoupling of 4.5 μm magnetic beads (M-450) on DSD wave pattern (green color).

D_{gap} : D_{s-wave} : D_{b-wave}	*Ratio by Bead diameter*	*Biased movement*		*Disaggregation*
		Mode 1	Mode 2	
4 μm: 5 μm: 10 μm	0.89 : 1.11 : 2.22	X	O	-
4 μm: 10 μm: 20 μm	0.89 : 2.22 : 4.44	O	X	X
4 μm: 15 μm: 30 μm	0.89 : 3.33 : 6.66	O	X	O
4 μm: 20 μm: 40 μm	0.89 : 4.44 : 8.88	O	X	X

Table S2. Condition for decoupling (green color) and inter-particle space control of 2.8 μm magnetic beads (M-280) on DSD wave pattern (blue color).

$D_{gap} : D_{s-wave} : D_{b-wave}$	*Ratio by Bead diameter*	*Biased movement*		*Disaggregation*
		Mode 1	Mode 2	
4 μm: 5 μm: 10 μm	1.43: 1.78: 3.57	Maintain initial direction		O
3 μm: 10 μm: 20 μm	1.07: 3.57: 7.14	O	X	O
3 μm: 20 μm: 40 μm	1.07: **7.14**: 14.29	O	X	X

Table S3. Condition for decoupling of 3.57 μm non-magnetic polymer beads (AP-35-10) on DSD wave pattern (green color).

$D_{gap} : D_{s-wave} : D_{b-wave}$	*Ratio by Bead diameter*	*Biased movement*		*Disaggregation*
		Mode 1	Mode 2	
4 μm : 10 μm : 20 μm	1.12 : 2.80 : 5.60	O	X	O
4 μm : 15 μm : 30 μm	1.12 : **4.20** : 8.40	O	X	O
5 μm : 15 μm : 30 μm	1.40 : **4.20** : 8.40	O	X	X

Table S4. Condition for decoupling of 6.72 μm non-magnetic polymer beads (AP-60-10) on DSD wave pattern (green color).

$D_{gap} : D_{s-wave} : D_{b-wave}$	*Ratio by Bead diameter*	*Biased movement*		*Disaggregation*
		Mode 1	Mode 2	
4 μm : 15 μm : 30 μm	0.59 : 2.23 : 4.46	Failed to cross the gap		-
5 μm : 15 μm : 30 μm	0.74 : 2.23 : 4.46	Failed to cross the gap		-
6 μm : 15 μm : 30 μm	0.89 : 2.23 : 4.46	O	X	O
6 μm : 20 μm : 40 μm	0.89 : 2.98 : 5.95	O	X	O

D_{gap}: gap size, D_{s-wave}: small wave diameter, D_{b-wave}: big wave diameter

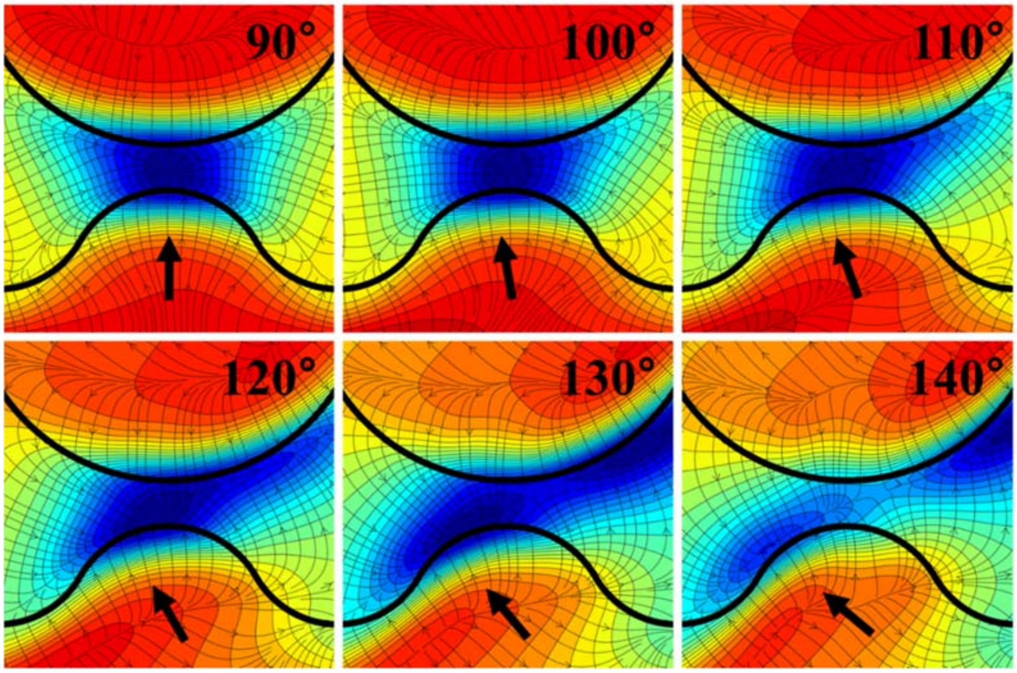

Figure S1. The potential energy distribution was created by the DSD pattern consisting of disks and the DSD wave-like pattern for continuous disaggregation and movement of the bead where their distributions are similar between 90 and 120°.

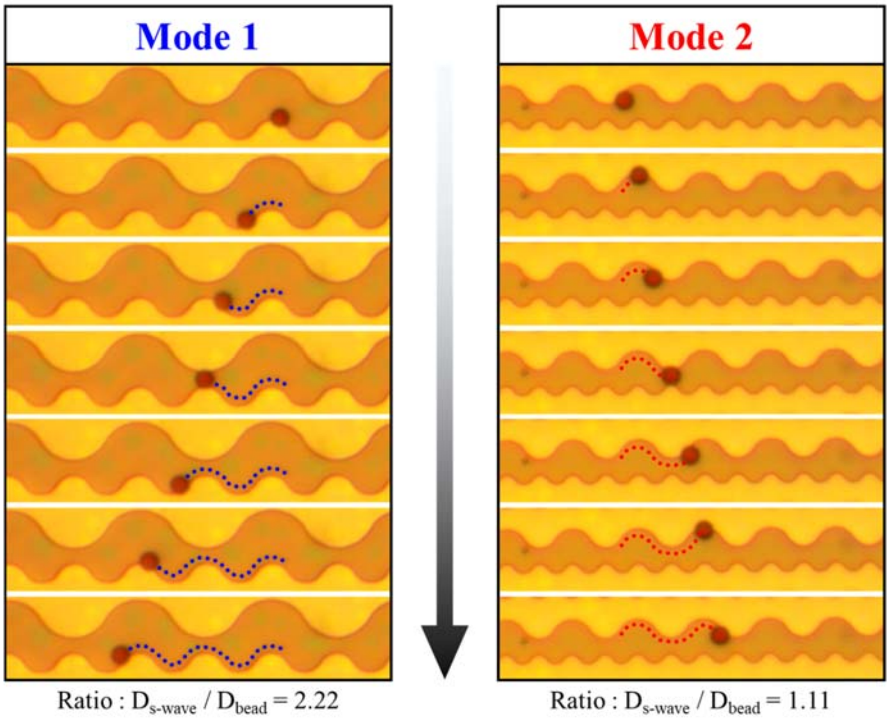

Figure S2. The "mode" depending on the ratio between pattern and bead diameter. In the "mode 1" bead is moving in lower track in $-$x-direction with the D_{bead} =4.5 μm, D_{s-wave} =10 μm, and D_{b-wave} =20 μm. With the D_{bead} =4.5 μm, D_{s-wave} =5 μm, D_{b-wave} =10 μm bead moves along the upper track in +x-direction, designated as "mode 2". If the diameter of the wavy disk is more than twice that of the bead diameter, i.e. D_{s-wave}/D_{bead} = 2.22, the bead moves along the small wave ("Mode 1"). If the ratio is less than twice (D_{s-wave}/D_{bead} = 1.11), the bead moved along a big wave ("Mode 2"). This is consistent with the simulation results.

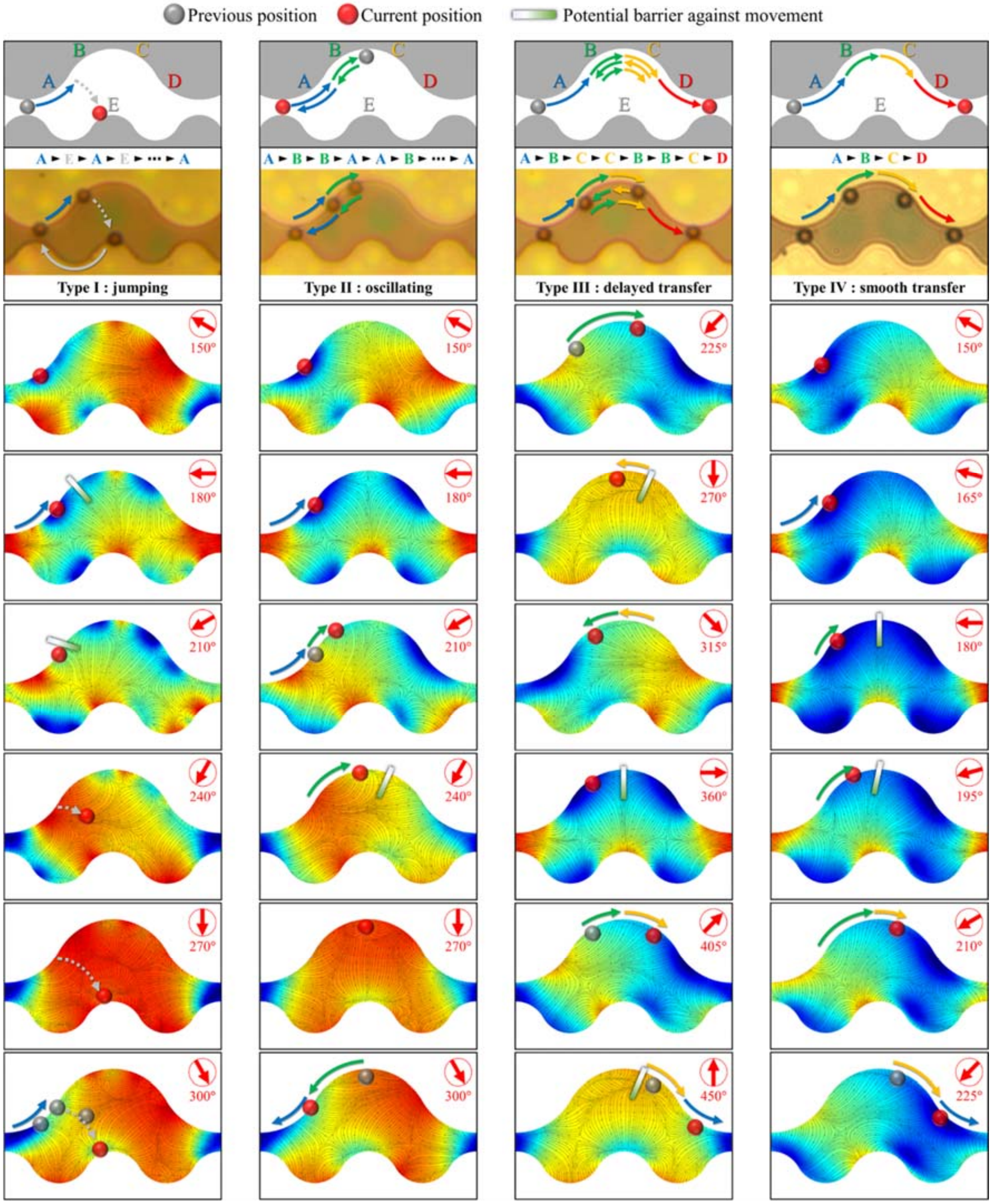

Figure S3. Bead motion by varying the field strength and frequency. The bead moved along the big wave by mode 2 or mode 1 (detached bead from the disaggregation process).

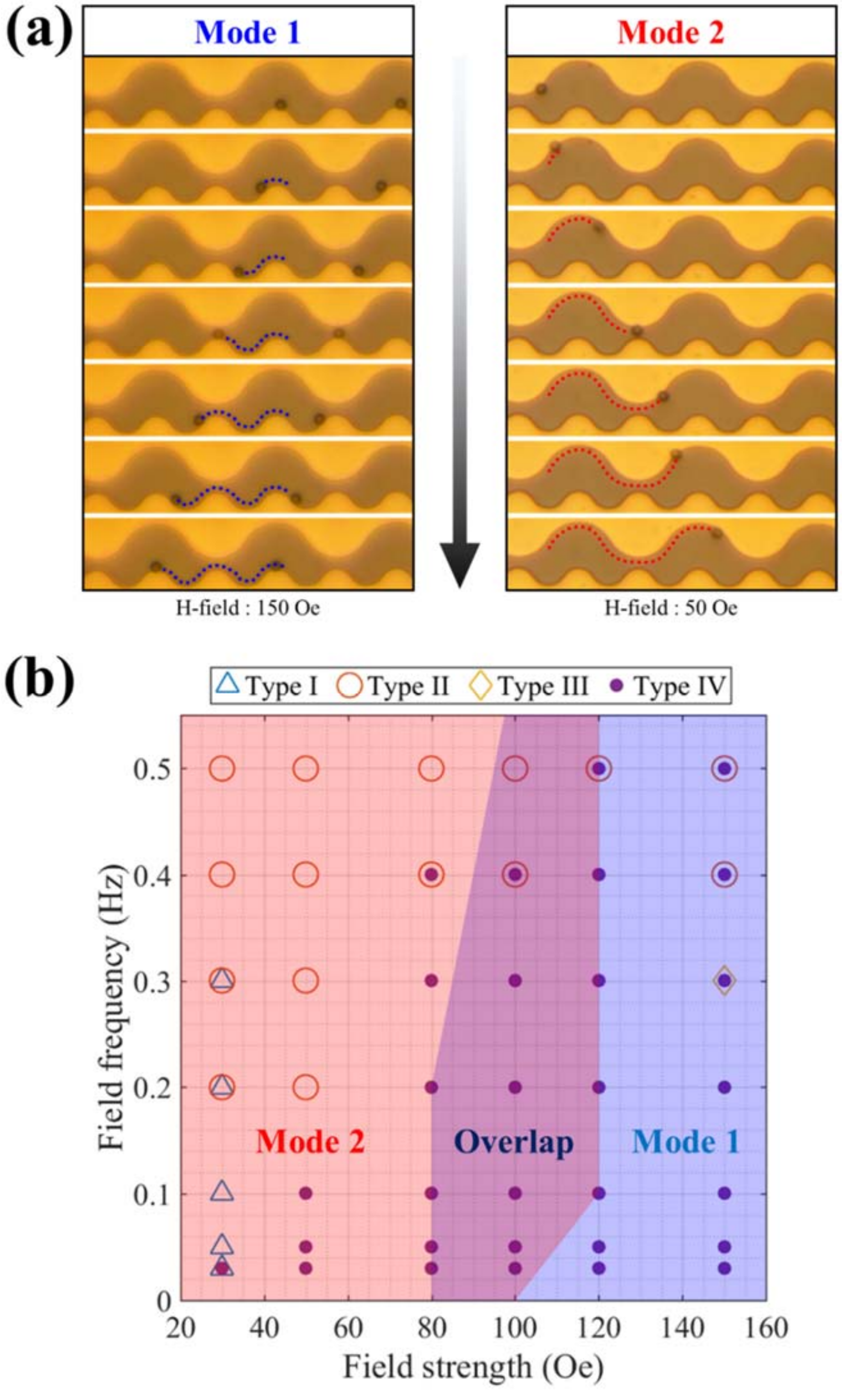

Figure 4. (a) Field strength dependence of "mode". In "mode 1" bead is moving in lower track in $-$x-direction with the D_{bead} = 2.8 μm, D_{s-wave} = 10 μm, and D_{b-wave} = 20 μm with the field strength and frequency of 150 Oe and 0.05 Hz. “Mode 2” also has the same condition as mode 1, but the field strength has changed to 50 Oe. (b) Phase diagram for movement of the bead as a function of field strength and frequency. Background color means the mode of the bead after mode switching. Symbols represent four types of movement of the bead along with the big wave as shown in Figure S2.

When the strength of the applied field exceeds 30 Oe and the frequency is below 0.5 Hz, “mode” switching occurs regardless of the field strength and frequency, where their field conditions will determine the preferred mode (**Movie 3**). Movement of the bead by mode 2 or detached bead from the disaggregation process by mode 1, four types of movement of the bead on the big wave are possible for field strengths between 0−150 Oe in the frequency range of 0.03−0.5 Hz (**Figure S2**). First, when the applied field is at 0 Oe $< H <$ 30 Oe and the frequency is below 0.3 Hz, jumping (Type I) of the bead from the big wave boundary occurs. Second, when 0 Oe $< H <$ 60 Oe and the frequency exceeds 0.2 Hz, oscillating (Type II) behavior of the bead between specific position occurs. At 60 Oe and above, the same oscillating phenomenon occurs when the frequency is greater than 0.4 Hz. Third, when the applied fields and frequency are around 150 Oe and 0.3 Hz respectively, delayed transfer of the bead (Type III) occurs. Fourth, while the field strength is over 30 Oe with certain corresponding frequencies, a complete smooth transfer (Type IV) of the bead occurs. Therefore, mode switching and the four types of movement on the big wave by mode 2 or detached bead from the disaggregation process by mode 1 have overlapping areas, as shown in the phase diagrams (**Figure S3, Movie 3**).

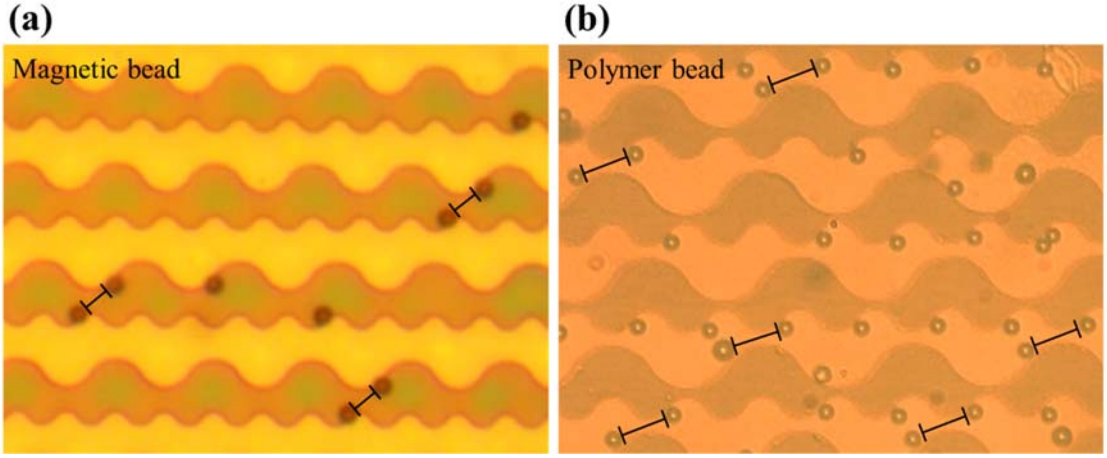

Figure S5. *Inter-particle distance control of multiple bead pairs*: Adjusted the spacing between each (a) magnetic and (b) non-magnetic polymer bead pairs. Both beads move in opposite directions with the magnetic beads along the cavity pattern and the polymer beads along on the magnetic pattern. The magnetic material and cavity are vertically symmetrical patterns to each other. Each bead pair maintains a constant distance.

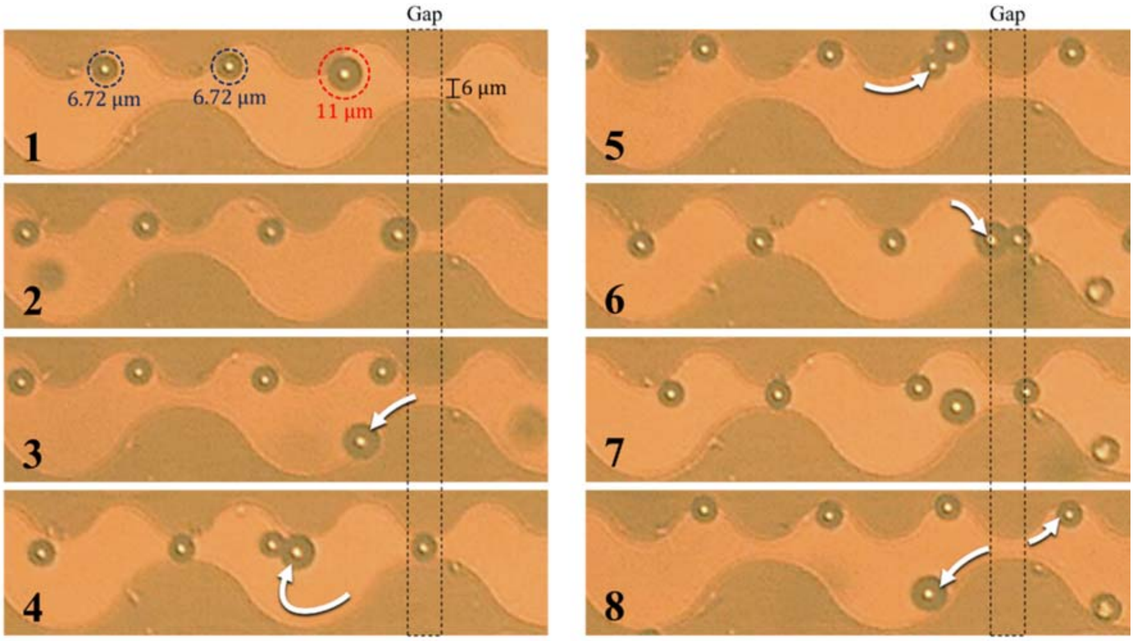

Figure S6. Non-magnetic polymer beads (6.72 and 11 µm diameter) disaggregation on the wave-like pattern (20 and 40 µm diameters wave with a gap of 6 µm) by applying a rotating magnetic field of 900 Oe. The aggregated polymer beads were split into single beads in the gap (decoupler) and then were moved in one direction with biased movement. The D_{gap}/D_{bead} for 6.72 and 11 µm diameter polymer beads are 0.89 and 0.54, respectively. Hence, as the "mode" operating range is 0.8−1.4, the 6.72 µm polymer beads are able to cross the gap whereas the 11 µm polymer unable to cross the gap.

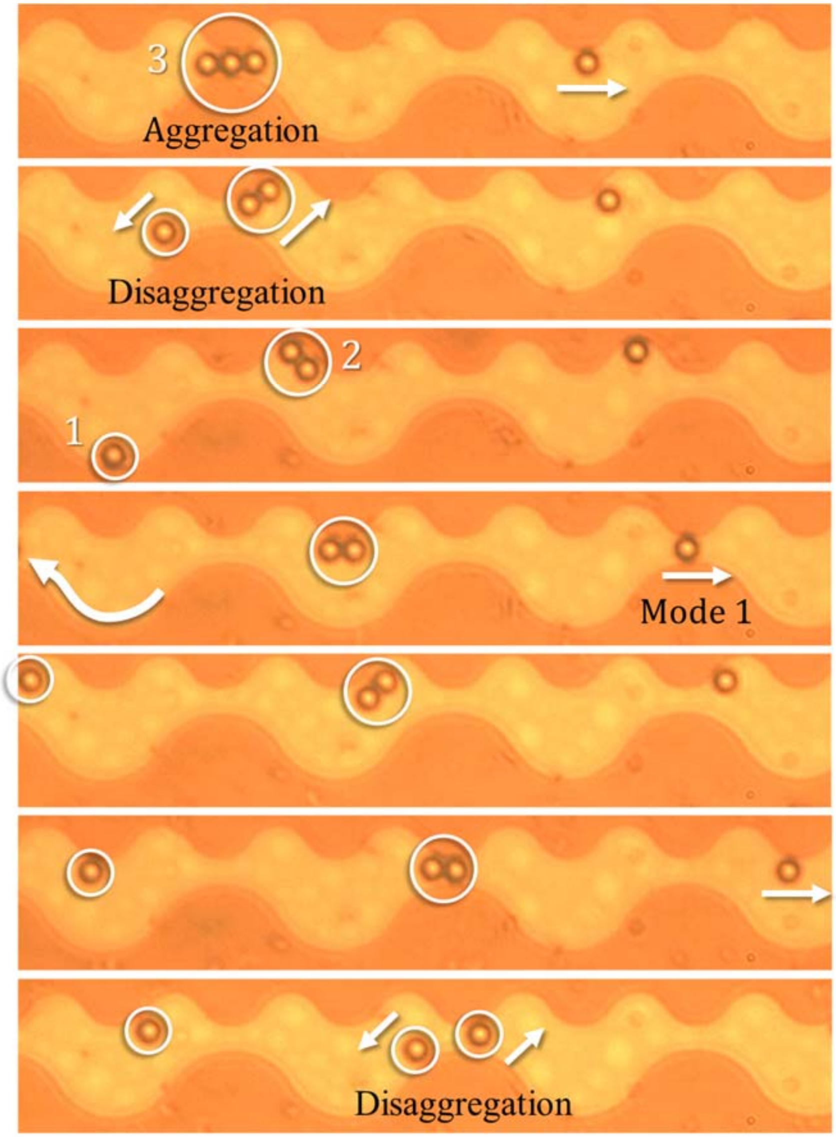

Figure S7. Disaggregation of non-magnetic polymer beads (3.57 μm diameter) at the decoupler of the wave-like pattern (10 and 20 μm diameters wave with a gap of 4 μm) by under a rotating magnetic field of 900 Oe. Its behavior is the same as a magnetic bead, but does not form a triangle because the dipole-dipole interaction between the beads is too small. The D_{gap}/D_{bead} for 3.57 μm diameter polymer beads is 1.12. Hence, the 3.57 μm polymer beads are able to cross the gap because the “mode” operating range is 0.8−1.4.

Supporting Movies

Movie 1: Movement of single with size dependence

Movie 2: Bead movement based on the barrier shape

Movie 3: Movement of single with field dependence

Movie 4: Disaggregation of the magnetic bead cluster into single bead at decoupler

Movie 5: The movement of bead that passes through each other at decoupler by maintaining initial direction

Movie 6: Simultaneous 2-dimensional multiple control of disaggregation and inter-particle distance

Movie 7: Disaggregation and selective movement of the non-magnetic beads at decoupler
